# Exploring brain functional connectivity in patients with taste loss: a pilot study

**DOI:** 10.1007/s00405-023-08019-4

**Published:** 2023-05-17

**Authors:** Yunmeng Zhu, Akshita Joshi, Divesh Thaploo, Thomas Hummel

**Affiliations:** Interdisciplinary Center Smell & Taste, Department of Otorhinolaryngology, Faculty of Medicine Carl Gustav Carus, Technische Universität Dresden, Fetscherstrasse 74, 01307 Dresden, Germany

**Keywords:** Taste loss, Gustation, fMRI, Functional connectivity

## Abstract

**Purpose:**

In a previous neuroimaging study, patients with taste loss showed stronger activations in gustatory cortices compared to people with normal taste function during taste stimulations. The aim of the current study was to examine whether there are changes in central-nervous functional connectivity in patients with taste loss.

**Methods:**

We selected 26 pairs of brain regions related to taste processing as our regions of interests (ROIs). Functional magnetic resonance imaging (fMRI) was used to measure brain responses in seven patients with taste loss and 12 healthy controls as they received taste stimulations (taste condition) and water (water condition). The data were analysed using ROI-to-ROI functional connectivity analysis (FCA).

**Results:**

We observed weaker functional connectivity in the patient group between the left and right orbitofrontal cortex in the taste condition and between the left frontal pole and the left superior frontal gyrus in the water condition.

**Conclusion:**

These results suggested that patients with taste loss experience changes of functional connectivity between brain regions not only relevant to taste processing but also to cognitive functions. While further studies are needed, fMRI might be helpful in diagnosing taste loss as an additional tool in exceptional cases.

## Introduction

The human sense of taste is important for the enjoyment of food and making food choices [1–3]. Taste loss often leads to negative effects on eating behaviour and nutritional status causing damages on human health [1, 4–6]. Clinically, the diagnosis of hypogeusia (partial taste loss) or ageusia (complete taste loss) largely depends on subjective complaints and psychophysical taste testing [7], which strongly relies on the cooperation of the patients. In cases of an inadequate ability to cooperate, e.g., in children or patients with cognitive impairments, or potential malingering concerning medicolegal contexts, psychophysical taste testing is unreliable [8]. Gustatory event-related potentials (GERPs) are less biased by the individuals’ beliefs and motives but due to the relatively complex technical prerequisites, the method is not widely used [9]. Histological investigations or contact endoscopy could help to examine morphological abnormalities of taste papillae/taste buds on the tongue [10, 11], but they are not standardized for diagnostic purposes. Magnetic resonance imaging (MRI) provides visualization of structural lesions of brain regions related to taste processing [12]. However, even in the absence of visible peripheral or central lesions [13–15], taste loss may persist and bother patients. Gustatory functional MRI (fMRI) provides a non-invasive way to examine gustatory function without a major bias in terms of cooperation from the patients. In addition, MRI scanners are widely available so that the technique could be easily applied in many different places.

To explore the potential use of fMRI in the diagnosis of taste loss, a previous gustatory fMRI study [16] was performed by our research group. In this study, eight patients with hypogeusia or ageusia and twelve healthy controls with normal taste function were recruited. The functional images of their brains when they were receiving taste stimulations were recorded using a 1.5 T scanner. We observed that the recognized primary and secondary taste cortices–insula cortex (IC) and orbital frontal cortex (OFC) [17] were activated by taste stimulations not only for most healthy participants but also for most patients with taste loss. There were considerable individual variations regarding the overall degree of activations and the sites of maximum activations. These results suggested that it is problematic to differentiate patients with taste loss from healthy controls based on gustatory functional MRI at an individual level. Interestingly, when doing group comparison, the patient group tended to show stronger activations in the IC and OFC, compared to the control group. This result was interpreted as patients with taste loss putting more efforts than controls into the processing of gustatory information.

In human brain, gustatory information is processed and transported in forms of neural networks of pathways arranged in series, in parallel and recurrently [18–21]. The temporal correlation of neuronal activation patterns of anatomically separated brain regions is defined as functional connectivity [22]. In the past years, increasing researchers have started to explore functional connectivity by calculating the correlation of time-series from different brain regions using fMRI [13, 14, 22]. The aim of the present study was to examine whether brain functional connectivity of patients with taste loss is different from that of healthy controls. We re-analysed the fMRI data collected in the previous study [16] using ROI-to-ROI (ROI: region of interest) Functional Connectivity Analysis (FCA), which allows us to see the functional connections between ROIs. So far, no study has investigated the functional connectivity of the brains of patients with taste loss. Hence, we took advantage of our previous data to explore this. Compared to healthy controls, in patients with taste loss we predicted significantly weaker functional connections between some ROIs, e.g., the primary and secondary taste cortices, IC and OFC [17].

## Methods

### Subjects

Seven patients with hypogeusia or ageusia (5 women, 2 men, mean age 56 years, age range 38–73 years, Table 1) and 12 healthy controls with normal taste function were included (6 women, 6 men, mean age 30 years, age range 21–51 years). All investigations were conducted in accordance with the Guidelines for Biomedical Studies Involving Human Subjects (Helsinki Declaration). The study protocol was approved by the Ethics Committee at the University Clinic “Gustav-Carl-Carus” of the “Technische Universitaet Dresden”. Written informed consent from all subjects was obtained before the experiment.

**Table 1:** Patients and clinical status

Patient no.	Gender	Age (years)	Gustatory function	Onset (months) prior to fMRI	Cause
2^a^	Woman	73	Hypogeusia	6	Infection of URT^b^
3	Woman	52	Hypogeusia	17	Head trauma
4	Man	50	Ageusia	54	Infection of URT
5	Woman	38	Hypogeusia	86	Unknown
6	Man	64	Hypogeusia	8	Head trauma
7	Woman	57	Ageusia	16	Infection of URT
8	Woman	59	Ageusia	12	Head trauma

^a^In the previous study, there were in total eight patients with taste loss. However, the data of one participant was damaged so that only 7 patients were included in the present study

^b^*URT* upper respiratory tract

These patients subjectively complained of taste loss and they were diagnosed with hypogeusia or ageusia based on a validated and reliable psychophysical taste test, the “taste strips” [23]. The duration of their taste loss varied between 6 and 86 months. In three patients, taste loss was reported after trauma, two after infections, and the remaining patient had no specific cause. Structural MR scans did not show any lesions of the brain related to the taste loss in any of the patients. Twelve healthy controls, who reported normal taste function, were ascertained as normogeusic with the identical “taste strips” test [23].

### Taste stimulation

Two taste qualities were used for taste stimulation: sweet and sour. Stimulants were administered in liquid form. The sweet stimulant was presented as a 2.92 mol/l sucrose solution, the sour one as a 0.21 mol/l citric acid solution. The solvent of the sweet/sour solution was water (ordered from Evian^®^, Danone Waters, Wiesbaden, Germany), which was also used as a control stimulation. Taste solutions were freshly prepared prior to each investigation.

Stimulants were delivered to the subject’s mouth using dedicated Teflon^®^ tubing fed through a small outlet in the wall of the scanner room. Three separate tubes for the respective stimulants (sweet, sour solution and water) were connected to one common mouthpiece which could easily be held by the subject’s lips and teeth. The other end of the tubing was connected to a three-way valve, which linked syringes, enabling the delivery and replenishment of the liquids, and blockage of flow from either end. Prior to the experiment, the tubes were filled with the respective stimulants by means of syringes. Stimulation was performed by releasing 0.1 ml liquid onto the subject’s tongue. Preliminary experiments on a small group of expert observers had ascertained that this amount (0.1 ml) of stimulant in the specific concentration produced a clear gustatory sensation and did not immediately evoke swallowing. Neither significant mechanical stimulation nor thermal stimulation was perceived in this amount (0.1 ml). Stimulants were presented at room temperature. In between stimulations, the subject’s mouth was rinsed with 2 ml of water. Subjects were instructed through message on a screen only to swallow during the “rinse” condition.

### Experimental design

**Fig. 1:**
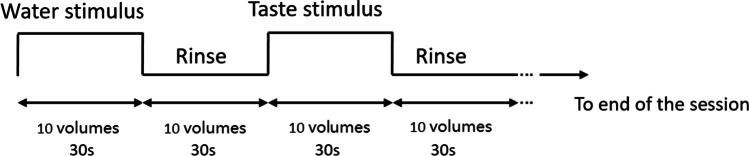
Experimental design. The first four conditions of one session are shown in the figure with 10 volumes (3 s for each volume) recorded for each condition

Each participant had one functional imaging investigation compromising four sessions (Table 2). In each session, there were three *experimental conditions*: 1. “Water” condition—water (0.1 ml) was presented; 2. “Rinse” condition—water (2 ml) was presented and subjects were only allowed to swallow in this condition; 3. “Taste” condition—sweet or sour solution (0.1 ml) was presented. The “Rinse” condition was established in order to prevent smearing effects on the tongue and enhance distinction of the taste/no-taste sensations. The “Rinse” condition was performed after each of the two main conditions (“Water” and “Taste” conditions), resulting in a basic sequential module of four conditions: Water (water 0.1 ml)—Rinse (water 2 ml)—taste (sweet/sour solution 0.1 ml)—Rinse (water 2 ml) (Fig. 1). This sequence of four conditions was repeated three times within each session, yielding a succession of 12 conditions (Fig. 1, Table 2). For each condition, 10 functional imaging volumes were obtained. With a repetition time of 3 s for each volume, the total scanning time of one complete fMRI investigation was 24 min. Within one session, only one type of taste quality was presented in “Taste” condition, either sweet or sour. Sweet and sour stimulants were presented in a randomized and alternating manner.

**Table 2:** Experimental design

Experimental condition	Scans (volumes)			
	Session 1 (sweet^a^)	Session 2 (sour)	Session 3 (sweet)	Session 4 (sour)
Water	0–9^b^	120–129	240–249	360–369
Rinse	10–19	130–139	250–259	370–379
Taste	20–29	140–149	260–269	380–389
Rinse	30–39	150–159	270–279	390–399
Water	40–49	160–169	280–289	400–409
Rinse	50–59	170–179	290–299	410–419
Taste	60–69	180–189	300–309	420–429
Rinse	70–79	190–199	310–319	430–439
Water	80–89	200–209	320–329	440–449
Rinse	90–99	210–219	330–339	450–459
Taste	100–109	220–229	340–349	460–469
Rinse	110–119	230–239	350–359	470–479

^a^Sweet and sour stimulants were presented in a randomized and alternating manner

^b^The number represented the number of volumes

### Data acquisition

Brain images were obtained by a Siemens-Sonata 1.5 T scanner (Siemens, Erlangen, Germany) with an eight-channel head coil. For functional imaging, a spin echo/echo planar imaging sequence, with echo time (TE) = 35 ms, repetition time = 3000 ms, flip angle = 90°, and 1 average. Slice thickness was 3 mm, slice spacing 3.75 mm. A total of 480 volumes were obtained in one run. Structural images were recorded using a T1 weighted sequence, with TR = 5.98 s, TE = 2.91 ms, 2 mm slice thickness, and 3 averages. One set consisted of 104 slices. In each subject, anatomy scans were acquired first, followed by the functional imaging run.

### Data analysis

ROI-to-ROI FCA was computed using the CONN toolbox [24], (http://www.nitrc.org/projects/conn), implemented in MATLAB. Preprocessing steps including realignment, coregistration/normalization, segmentation, outlier identification and smoothing, and de-noising steps which aim to remove possible confounds in the BOLD signal, including motion, physiological and other noise sources were all done using the CONN toolbox [24].

After pre-setting region of interests (ROIs), a General Linear Model (GLM) was used to calculate correlations of the mean BOLD time-series between each two different ROIs at the single-subject level, resulting ROI-to-ROI functional connectivity matrices consisting Fisher-transformed bivariate correlation coefficients (z-scores) between each two different ROIs (https://web.conn-toolbox.org/fmri-methods/connectivity-measures/roi-to-roi) in two different task conditions. Task conditions include “Taste condition” and “Water condition” (Fig. 1, Table 2). Both sweet and sour taste stimulants were evaluated as one common “taste condition”. Group analysis was then performed using a two-sample *t* test to uncover differences in functional connections between the patient and control groups in both conditions. Age and sex of participants were introduced as covariates into the analysis. Connection threshold *p* < 0.05 (p-FWE corrected) was regarded as significant.

The pre-set ROIs in the present study included right and left IC [16, 17, 25, 26], operculum [25, 26], OFC [25, 26], cingulate [25, 26], amygdala [25, 27], thalamus [26, 28], cerebellum [25], temporal pole [25] and putamen [25], identified as relevant regions with respect to taste cerebral processing by previous studies. We also added ROIs related to frontal cortices considering their roles in modulating gustatory processing [19]. Because of the close relation between gustation and olfaction we added the piriform cortex (PFC), which is considered to be a significant part of the primary olfactory cortex [17]. The ROIs of OFC and PFC were provided by Fjaeldstad et al. [29]. The remaining ROIs were chosen from the FSL Harvard–Oxford Atlas and the AAL atlas provided by the software [30], resulting in a total of 52 ROIs (26 pairs) in the FCA (Table 3).

**Table 3:** Selected ROIs for FCA

ROIs (Region of interests)
ROIs provided by the CONN (the FSL Harvard–Oxford atlas and the AAL atlas)
Insular Cortex (r & l)^a^
Frontal Operculum Cortex (r & l)
Parietal Operculum Cortex (r & l)
Cingulate Gyrus, anterior division
Cingulate Gyrus, posterior division
Amygdala (r & l)
Thalamus (r & l)
Putamen (r & l)
Cerebellum Crus 1–10 (r & l)
Temporal Pole (r & l)
Postcentral Gyrus (r & l)
Frontal Pole (r & l)
Superior Frontal Gyrus (r & l)
Middle Frontal Gyrus (r & l)
Inferior Frontal Gyrus, pars triangularis (r & l)
Inferior Frontal Gyrus, pars opercularis (r & l)
ROIs provided by Fjaeldstad et al.
Orbitofrontal cortex (r & l)
Prefrontal cortex (r & l)

^a^r & l: right and left

## Results

### Group level

**Fig. 2:**
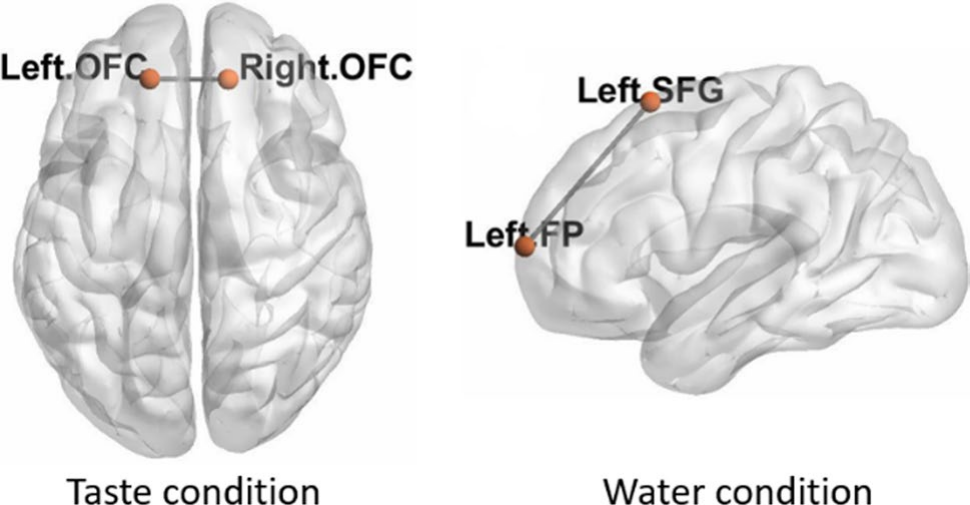
Two functional connections in the patient group were significantly weaker than in the control group. One was between the right and the left orbital frontal cortex (OFC) in the taste condition [T (17) = 6.79, connection threshold: *p* < 0.05, p-FWE corrected, left panel], another one was between the left Frontal Pole (FP) and the left Superior Frontal Gyrus (SFG) in the water condition [T (17) = 5.16, connection threshold: p < 0.05, p-FWE corrected, right panel]

In the taste condition, the patient group showed significantly weaker functional connectivity between left OFC (lOFC) and right OFC (rOFC) compared to the control group [T (17) = 6.79, connection threshold: *p* < 0.05, p-FWE corrected, Fig. 2 left panel].

For the water condition, the patient group showed significantly weaker functional connectivity between left frontal pole (lFP) and left superior frontal gyrus (lSFG) in comparison to the control group [T(17) = 5.16, connection threshold: *p* < 0.05, p-FWE corrected, Fig. 2 right panel].

The functional connectivity of ROIs was also compared between taste and water conditions but there were no significant differences for all participants.

### Individual level

**Fig. 3:**
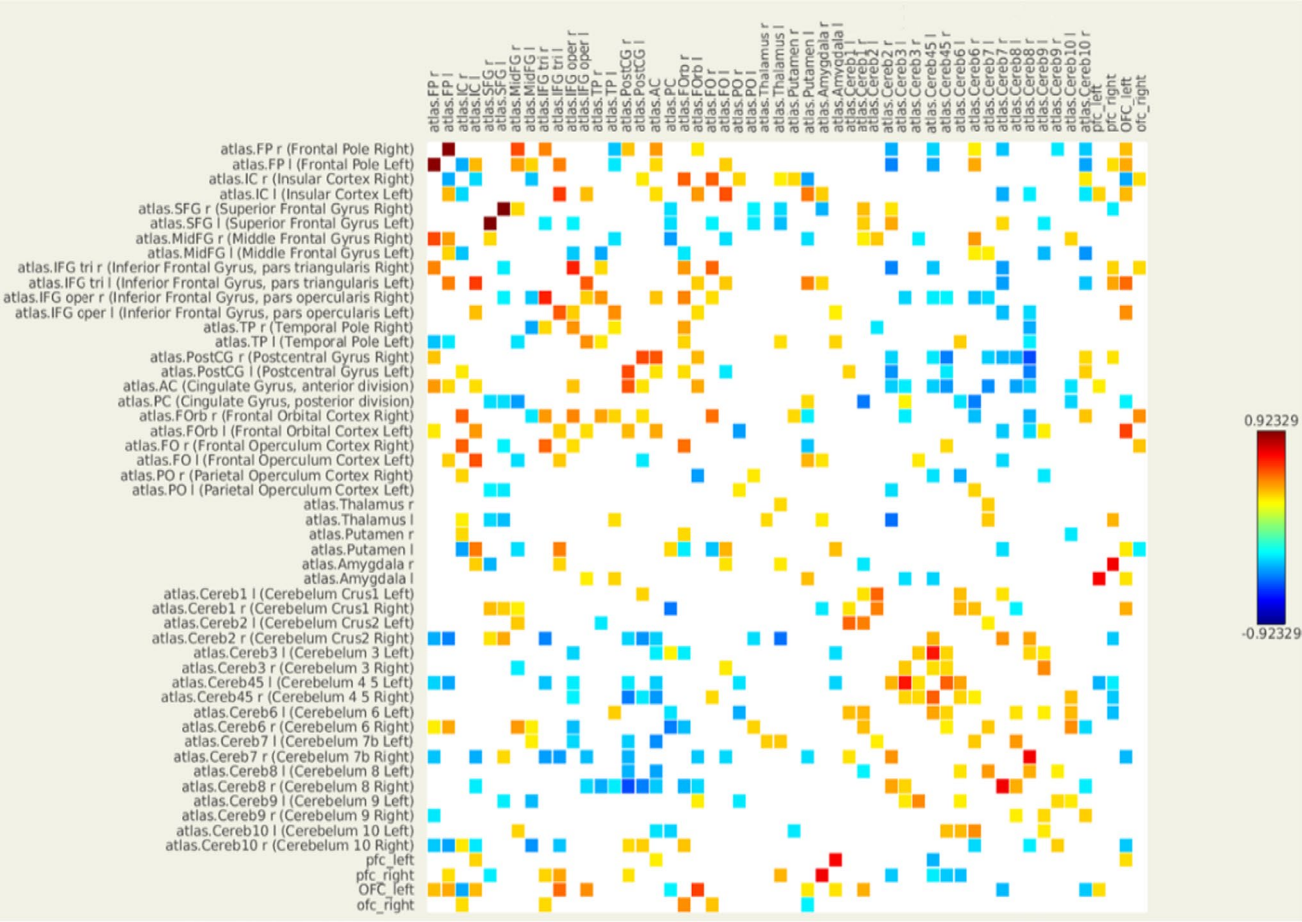
An ROI-to-ROI connectivity (RRC) matrix of one single-subject of taste condition created by the CONN toolbox. Each element in an RRC matrix is defined as the Fisher-transformed bivariate correlation coefficient (z-score) between a pair of ROI BOLD time series. We pre-defined 52 regions of interest (ROIs) and the calculated z-scores form a 52-by-52 matrix (the z-score was not calculated between one ROI and itself). The squares are shown/coloured when z-scores were above + 0.25 or below − 0.25

On an individual level, each participant had a unique pattern of ROI-to-ROI connectivity (RRC) matrix containing Fisher-transformed bivariate correlation coefficients (z-scores) between every two different ROIs. Figure 3 is an example of the RRC matrix of one single subject of taste condition. On the individual level, RRC can be thresholded based on z-scores but only for display purposes and it is not supported by any form of statistical inference as reported by the CONN forum (https://www.nitrc.org/forum/message.php?msg_id=5149).

As *group* analysis showed two pairs of functional connections (lOFC–rOFC and lFP–lSFG) that were significantly different between patient and control group, we checked these two pairs of connections in individual RRC matrix. We set z-score > 0.25 (0.25 is the default value set by the CONN toolbox) as a threshold to display the matrix. As shown in Table 4, in the water condition, z-scores corresponding to the lFP–lSFG connection were more than 0.25 in 9 out of 12 healthy controls but were less than or equal to 0.25 in all 7 patients. In taste condition, for all patients and most healthy controls (9 out of 12), the z-scores corresponding to the lOFC–rOFC connection were less than or equal to 0.25.

**Table 4:** Two functional connections in individual level

	Taste condition	Water condition
	lOFC and rOFC	lFP and lSFG
Patient no.		
2	–	–
3	–	–
4	–	–
5	–	–
6	–	–
7	–	–
8	–	–
Control no.		
1	–	+
2	+	–
3	–	+
4	–	+
5	+	–
6	–	+
7	–	+
8	–	–
9	–	+
10	–	+
11	–	+
12	+	+

Two functional connections in individual level. “ + ” means the z-score is more than 0.25 while “–” means z-scores is less than or equal to 0.25. Z-score equals to Fisher-transformed bivariate correlation coefficients between the left and right orbital frontal cortex (OFC) in taste condition, or between the left frontal pole (lFP) and the left superior frontal gyrus (lSFG) in water condition

## Discussion

On a group level, a weaker functional connection between right and left OFC was observed in patients with taste loss compared to healthy controls during taste stimulations. In primates, OFC receives direct inputs from the primary taste cortex—IC [31] and neurons in the OFC can respond to the prototypical tastes [32, 33]. Human neuroimaging studies also showed that the OFC can be activated by gustatory stimuli [34–36]. Hence, the OFC is considered to contain the secondary taste cortex in humans [17]. It also plays an important role in integrating gustation with retronasal olfaction and oral somatosensation into a “flavor” [37]. The right and left OFCs are anatomically separated in two hemispheres. In the previous study [16] of our laboratory, when participants receiving taste stimulations, activations of the right and left OFCs in the patient group tended to be stronger than that in control group. Interestingly, at the same situation, the functional connectivity between right and left OFCs was weaker in patient group compared to control group based on the FCA in the present study.

To our knowledge, no other study has investigated how brain functional connectivity changes after a long-term taste loss. However, there was a study investigating the effects of chronic *peripheral* olfactory loss on brain functional connectivity where patients with long-term peripheral olfactory loss and healthy controls were asked to do a sniffing task in the MRI scanner. The FCA revealed that compared to healthy controls, patients with olfactory loss showed a decrease in functional connectivity [38] involving the anterior prefrontal cortex, the anterior cingulate cortex, the entorhinal cortex and the cerebellum [38]. In other words, long-term peripheral olfactory loss is associated with decreased functional connectivity among the brain regions relevant to olfactory processing. This is consistent with what we have found on our patients with long-term taste loss.

Generally, there were two types of task conditions in the present study that is taste condition and water condition. Taste condition is a condition that participants were receiving sweet or sour taste stimulations (0.1 ml sweet/sour solutions). Water condition is a condition that participants were perceiving purely water (0.1 ml water). Importantly, water has been mentioned as an independent taste modality [39]. The functional connectivity of ROIs has been compared between taste and water conditions but there were no significant differences between two conditions at a group level. This might be because the sample size was too small to show the significant difference; or the difference between cerebral processing of taste solutions and that of pure water does not manifest itself in the level of functional connectivity.

Interestingly, we observed, when participants perceiving purely water (in water condition), a weaker functional connectivity between the left frontal pole (lFP) and the left superior frontal gyrus (lSFG) in the patient group compared to the control group. FP contains areas associated with many higher cognitive functions such as drawing analogies and making plans [40, 41]. The SFG is generally thought as a core brain region in cognitive control systems [42]. Cognitive functions are expected to modulate taste-related activations in gustatory cortices [19]. The decrease in functional connectivity between brain regions relevant to cognition may contribute to the perceived taste loss. Several studies [43–45] have demonstrated that people with cognitive impairments or with dementia in the early stage exhibited significant impairments of taste sensitivity in comparison to age-matched healthy controls. These findings suggest a close relation between taste function and cognition. Unfortunately, the cognitive abilities of our participants were not evaluated at that time. Nevertheless, the finding seems to emphasize the association between taste function and cognition. Cognitive functions should receive more attention when patients complain about taste loss, especially in idiopathic taste loss. In this perspective, fMRI could be an available tool to follow up patients and evaluate brain changes at the level of functional connectivity.

At an individual level, each participant exhibited a unique pattern of ROI-to-ROI functional connectivity (RRC) matrix. This was true for patients and controls. This individual variation was expected because sensory systems are highly plastic [38, 46] at both cellular [47] and cognitive levels [48], which relates to learning/training experiences [49, 50]. As mentioned above, at a group level, we found a weaker functional connectivity between the lFP and the lSFG in the patient group compared to the control group. In individual level, we found that z-scores corresponding to the lFP–lSFG connection were more than 0.25 in 9 out of 12 healthy controls but were less than or equal to 0.25 in all patients with taste loss (Table 4). This provides an impression that there might be a useful criterion regarding the z-scores that could differentiate patients with taste loss from healthy controls. This impression suggests the possibility that fMRI might help diagnosing taste loss as an additional tool in future by focusing on specific functional connections.

However, the shortcoming of the present study is not only the small sample size but also the uncertainty and inconsistency regarding the aetiology of taste loss. Regarding the seven patients in our study, three patients claimed that their taste losses started after head traumas. However, their structural MR scans did not show any lesions that could be related to the taste loss in the brain. Three patients claimed that their taste loss began after infections of the upper respiratory tract (also with no lesions visible in structural brain imaging). The remaining patient had idiopathic taste loss. The common characteristic of these patients was the long-term taste loss and the impaired ability to identify taste qualities based on psychophysical taste testing. So far, we could only suggest that symptoms of taste loss as well as impaired taste identification ability are associated with decreased central functional connectivity between some brain regions. We cannot make certain whether the symptoms of taste loss cause the decreased functional connectivity of the brain regions or the other way around. In addition, the sample size of the current study was too small. Introducing age and gender as covariates further reduced the power of analysis. Hence, we framed the study as exploratory and hope these observations might provide some useful references for other researchers to design related studies. Future studies with a larger sample size, age/gender-matched controls and with subgroups, e.g., (1) a subgroup including patients with long-term peripheral taste loss and (2) a subgroup including patients with idiopathic taste loss (possibly related to earlier central cognitive damages), should elucidate the possible causal relationship of the decreased functional connectivity of brain regions and the symptom of taste loss.

## Conclusion

Patients with taste loss appear to have central functional changes in terms of decreased functional connectivity between brain regions not only relevant for taste processing but also for cognition. While further studies are needed, fMRI might be helpful in diagnosing taste loss as an additional tool in some exceptional cases.

## Data Availability

The datasets generated during and/or analysed during the current study are available from the corresponding author on reasonable request.

## References

[1] Clark JE (1998). Taste and flavour: their importance in food choice and acceptance. Proc Nutr Soc.

[2] Kabir A, Miah S, Islam A (2018). Factors influencing eating behavior and dietary intake among resident students in a public university in Bangladesh: a qualitative study. PLoS ONE.

[3] Stok FM, Renner B, Clarys P, Lien N, Lakerveld J, Deliens T (2018). Understanding eating behavior during the transition from adolescence to young adulthood: a literature review and perspective on future research directions. Nutrients.

[4] Schiffman SS (1983). Taste and smell in disease. N Engl J Med.

[5] Sergi G, Bano G, Pizzato S, Veronese N, Manzato E (2017). Taste loss in the elderly: possible implications for dietary habits. Crit Rev Food Sci Nutr.

[6] Xue Y, Wen Q, Xu C, Zhang X, Zeng J, Sha AM, Lan C, Li L, Wang H, Yang X (2020). Elevated salt taste threshold is associated with increased risk of coronary heart disease. J Cardiovasc Trans Res.

[7] Zhu Y, Hummel T (2021). Assessment of taste function. Handb Exp Pharmacol.

[8] Hummel T, Hummel C, Welge-Luessen A, Welge-Lüssen A, Hummel T (2004). Assessment of olfaction and gustation. Management of smell and taste disorders.

[9] Hummel T, Genow A, Landis BN (2010). Clinical assessment of human gustatory function using event related potentials. J Neurol Neurosurg Psychiatry.

[10] Walliczek-Dworschak U, Schöps F, Feron G, Brignot H, Hähner A, Hummel T (2017). Differences in the density of fungiform papillae and composition of saliva in patients with taste disorders compared to healthy controls. Chem Senses.

[11] Srur E, Pau HW, Just T (2011). Changes in taste bud volume during taste disturbance. Auris Nasus Larynx.

[12] Abolmaali N, Welge-Lüssen A, Hummel T (2004). Structural imaging in chemosensory dysfunction. Management of smell and taste disorders.

[13] Arey L, Tremaine M, Monzingo F (1935). The numerical and topographical relations of taste buds to human circumvallate papillae throughout the life span. Anat Rec.

[14] Pavlidis P, Gouveris H, Anogeianaki A, Koutsonikolas D, Anogianakis G, Kekes G (2013). Age-related changes in electrogustometry thresholds, tongue tip vascularization, density, and form of the fungiform papillae in humans. Chem Senses.

[15] Just T, Pau HW, Witt M, Hummel T (2006). Contact endoscopic comparison of morphology of human fungiform papillae of healthy subjects and patients with transected chorda tympani nerve. Laryngoscope.

[16] Hummel C, Frasnelli J, Gerber J, Hummel T (2007). Cerebral processing of gustatory stimuli in patients with taste loss. Behav Brain Res.

[17] Rolls ET (2019). Taste and smell processing in the brain. Handb Clin Neurol.

[18] Katz DB, Nicolelis MA, Simon SA (2002). Gustatory processing is dynamic and distributed. Curr Opin Neurobiol.

[19] Jones LM, Fontanini A, Katz DB (2006). Gustatory processing: a dynamic systems approach. Curr Opin Neurobiol.

[20] Lemon CH, Katz DB (2007). The neural processing of taste. BMC Neurosci.

[21] Fonseca E, de Lafuente V, Simon SA, Gutierrez R (2018). Sucrose intensity coding and decision-making in rat gustatory cortices. Elife.

[22] van den Heuvel MP, Hulshoff Pol HE (2010). Exploring the brain network: a review on resting-state fMRI functional connectivity. Eur Neuropsychopharmacol.

[23] Landis BN, Welge-Luessen A, Brämerson A, Bende M, Mueller CA, Nordin S, Hummel T (2008). “Taste Strips” - a rapid, lateralized, gustatory bedside identification test based on impregnated filter papers. J Neurol.

[24] Whitfield-Gabrieli S, Nieto-Castanon A (2012). Conn: a functional connectivity toolbox for correlated and anticorrelated brain networks. Brain Connect.

[25] Small DM, Gregory MD, Mak YE, Gitelman D, Mesulam MM, Parrish T (2003). Dissociation of neural representation of intensity and affective valuation in human gustation. Neuron.

[26] Veldhuizen MG, Albrecht J, Zelano C, Boesveldt S, Breslin P, Lundström JN (2011). Identification of human gustatory cortex by activation likelihood estimation. Hum Brain Mapp.

[27] Hoogeveen HR, Dalenberg JR, Renken RJ, ter Horst GJ, Lorist MM (2015). Neural processing of basic tastes in healthy young and older adults—an fMRI study. Neuroimage.

[28] Yeung AW, Tanabe HC, Suen JL, Goto TK (2016). Taste intensity modulates effective connectivity from the insular cortex to the thalamus in humans. Neuroimage.

[29] Fjaeldstad A, Fernandes HM, Van Hartevelt TJ, Gleesborg C, Møller A, Ovesen T, Kringelbach ML (2017). Brain fingerprints of olfaction: a novel structural method for assessing olfactory cortical networks in health and disease. Sci Rep.

[30] Tzourio-Mazoyer N, Landeau B, Papathanassiou D, Crivello F, Etard O, Delcroix N, Mazoyer B, Joliot M (2002). Automated anatomical labeling of activations in SPM using a macroscopic anatomical parcellation of the MNI MRI single-subject brain. Neuroimage.

[31] Baylis LL, Rolls ET, Baylis GC (1995). Afferent connections of the caudolateral orbitofrontal cortex taste area of the primate. Neuroscience.

[32] Rolls ET, Yaxley S, Sienkiewicz ZJ (1990). Gustatory responses of single neurons in the caudolateral orbitofrontal cortex of the macaque monkey. J Neurophysiol.

[33] Baylis LL, Rolls ET (1991). Responses of neurons in the primate taste cortex to glutamate. Physiol Behav.

[34] Zald DH, Hagen MC, Pardo JV (2002). Neural correlates of tasting concentrated quinine and sugar solutions. J Neurophysiol.

[35] O'Doherty J, Rolls ET, Francis S, Bowtell R, McGlone F (2001). Representation of pleasant and aversive taste in the human brain. J Neurophysiol.

[36] Rolls ET (2008). Functions of the orbitofrontal and pregenual cingulate cortex in taste, olfaction, appetite and emotion. Acta Physiol Hung.

[37] Small DM, Bender G, Veldhuizen MG, Rudenga K, Nachtigal D, Felsted J (2007). The role of the human orbitofrontal cortex in taste and flavor processing. Ann N Y Acad Sci.

[38] Kollndorfer K, Jakab A, Mueller C, Trattnig S, Schöpf V (2015). Effects of chronic peripheral olfactory loss on functional brain networks. Neuroscience.

[39] Rosen AM, Roussin AT, Di Lorenzo PM (2010). Water as an independent taste modality. Front Neurosci.

[40] Fuster JM (2002). Frontal lobe and cognitive development. J Neurocytol.

[41] Bludau S, Eickhoff SB, Mohlberg H, Caspers S, Laird AR, Fox PT, Schleicher A, Zilles K, Amunts K (2014). Cytoarchitecture, probability maps and functions of the human frontal pole. Neuroimage.

[42] Niendam TA, Laird AR, Ray KL, Dean YM, Glahn DC, Carter CS (2012). Meta-analytic evidence for a superordinate cognitive control network subserving diverse executive functions. Cogn Affect Behav Neurosci.

[43] Contri-Degiovanni PV, Degiovanni GC, Ferriolli E, da Costa Lima NK, Moriguti JC (2020). Impact of the severity of dementia due to Alzheimer's disease on the gustatory sensitivity of older persons. Aging Clin Exp Res.

[44] Steinbach S, Hundt W, Vaitl A, Heinrich P, Förster S, Bürger K, Zahnert T (2010). Taste in mild cognitive impairment and Alzheimer's disease. J Neurol.

[45] Sakai M, Kazui H, Shigenobu K, Komori K, Ikeda M, Nishikawa T (2017). Gustatory dysfunction as an early symptom of semantic dementia. Dement Geriatr Cogn Disord Extra.

[46] Goldstone RL (1998). Perceptual learning. Annu Rev Psychol.

[47] Cadiou H, Aoudé I, Tazir B, Molinas A, Fenech C, Meunier N, Grosmaitre X (2014). Postnatal odorant exposure induces peripheral olfactory plasticity at the cellular level. J Neurosci.

[48] Bende M, Nordin S (1997). Perceptual learning in olfaction: professional wine tasters versus controls. Physiol Behav.

[49] Gilbert CD, Sigman M (2007). Brain states: top-down influences in sensory processing. Neuron.

[50] Kollndorfer K, Fischmeister FPS, Kowalczyk K, Hoche E, Mueller C, Trattnig S, Schöpf V (2015). Olfactory training induces changes in regional functional connectivity in patients with long-term smell loss. NeuroImage Clin.

